# High-Payload and Secure Data Hiding for Medical Images in IoMT-Based eHealth Systems

**DOI:** 10.3390/s26103032

**Published:** 2026-05-11

**Authors:** Yichen Wang, Yijie Lin, Ching-Chun Chang, Chin-Chen Chang, Wu-Yuin Hwang

**Affiliations:** 1 School of Management Science and Engineering, Shandong Technology and Business University, Yantai 264005, China; 2023210315@sdtbu.edu.cn; 2 Department of Information Engineering and Computer Science, Feng Chia University, Taichung 40724, Taiwan; 3 National Institute of Informatics, Tokyo 101-8430, Japan; ccchang@nii.ac.jp; 4 Department of Computer Science and Information Engineering, College of Science and Engineering, National Dong Hwa University, Hualien 97401, Taiwan; wyhwang1206@gmail.com; 5 Institute of Network Learning Technology, National Central University, Taoyuan 32001, Taiwan

**Keywords:** Absolute Moment Block Truncation Coding, block classification, data hiding, Internet of Medical Things, puzzle matrix

## Abstract

With the rapid advancement of the Internet of Medical Things (IoMT), the efficient transmission and management of large-scale medical images in bandwidth- and resource-constrained networks remain critical challenges. This paper proposes a high-payload data hiding method in Absolute Moment Block Truncation Coding (AMBTC)-compressed medical images based on block classification. Image blocks are categorized into flat, smooth, and complex types according to the difference between high and low values, and adaptive embedding and extraction strategies are applied to each type. The proposed method integrates secret data into the compression framework, thereby enhancing efficiency while maintaining visual quality. Experimental results demonstrate an average efficiency of 59% and an average PSNR of approximately 30 dB. Furthermore, visual and structural evaluations indicate that the proposed method effectively preserves textures and boundaries. These results confirm the feasibility of integrating high-payload data hiding into AMBTC compression for efficient medical image storage and transmission in IoMT environments.

## 1. Introduction

With the rapid development of the Internet of Things (IoT) and 5G communication technologies, smart healthcare systems such as telemedicine and eHealth have been widely adopted, fundamentally transforming traditional medical practices [1,2]. In modern remote healthcare scenarios, medical IoT devices, collectively referred to as the Internet of Medical Things (IoMT) [3,4], generate and transmit large volumes of medical images daily, including X-rays, ultrasound scans, magnetic resonance imaging (MRI), and computed tomography (CT) images. These devices often operate under constrained computational and memory resources while processing multimodal image data. Efficient compression of medical images prior to transmission is therefore essential to reduce network latency, alleviate bandwidth constraints, and minimize cloud storage requirements [5]. Among existing compression techniques, Absolute Moment Block Truncation Coding (AMBTC) [6] is particularly suitable for resource-constrained IoMT devices due to its low computational complexity, minimal memory requirements, and satisfactory image reconstruction quality.

**Figure 1:**
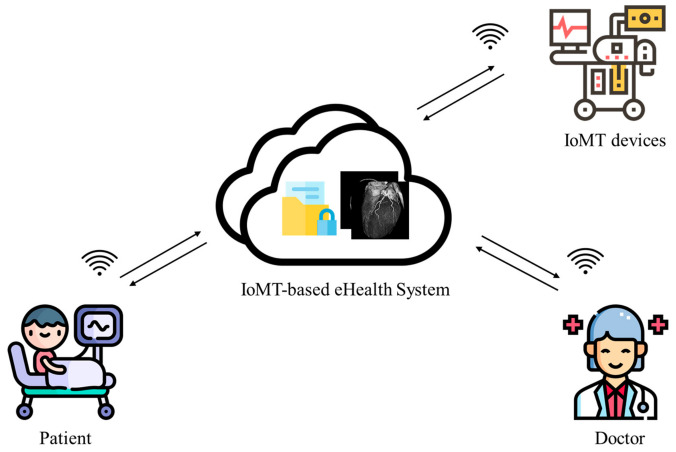
Application scenario of an IoMT-based eHealth system.

The remote transmission of medical images raises significant security and privacy concerns, as these images are associated with highly sensitive electronic patient records or personal health information. As shown in Figure 1, in an IoT-based eHealth system, IoMT devices first collect medical images or physiological data from patients and transmit them to a cloud server for storage and processing. Authorized medical professionals and patients can subsequently retrieve these records, with professionals using them for remote diagnosis and patients accessing their own health data. To facilitate secure transmission, various methodologies, such as data encryption and private channels, have been employed to ensure confidentiality. Among these, data hiding techniques [7,8,9,10,11,12] have been widely investigated as an approach for preserving the intrinsic association between images and patient information. By embedding patient data into medical images in an imperceptible manner, data hiding enables covert transmission and mitigates the risks of data loss, separation, and tampering.

Data hiding has been widely explored in the compression domain [13,14], including AMBTC [15,16], vector quantization (VQ) [17], and JPEG [18,19,20]. AMBTC-based data hiding is particularly suitable for IoMT scenarios because it enables devices to embed information during image compression. Researchers have focused on maximizing the payload while minimizing perceptual distortion by modifying the AMBTC-compressed code, including the quantized high and low values and the bitmap. Early work by Ou and Sun [21] in 2015 proposed a minimal-distortion, high-payload AMBTC-based image data hiding method. In 2019, Kumar et al. [22] enhanced AMBTC-based hiding by integrating Hamming distance and pixel value differencing mechanisms to achieve a better trade-off between payload and image quality. In the same year, Chang et al. [23] employed a block classification strategy with replacement, matrix coding, and block-specific symmetric quantization. In 2023, Chen et al. [24] adopted the block classification approach and introduced a gradient-based compression method compatible with AMBTC. In 2024, Lin et al. [25] introduced reference matrices to improve efficiency and proposed a variant that does not increase file size. Between 2024 and 2025, Lin et al. [26,27,28] extended the block classification method to four types, further balancing visual quality and payload.

Inspired by previous studies, this paper proposes a high-payload data hiding method for medical images compressed using AMBTC. Image blocks are first classified into three types called flat blocks, smooth blocks, and complex blocks based on the difference between high and low pixel values and predefined thresholds. Customized embedding strategies are applied to each block type. Flat blocks use only the block mean for reconstruction, freeing space for secret data. Smooth blocks retain essential pixel information while compressing the bitmap, allowing additional data to be embedded. Complex blocks preserve all intrinsic information and embed data using a puzzle matrix [29] and the storage order of pixel values, ensuring minimal visual distortion. The main contributions of this paper are summarized as follows:

- A novel data hiding method for AMBTC-compressed images in IoMT is proposed, where block classification ranges are adjusted according to thresholds.
- Residual analysis and edge intersection maps demonstrate that the structural integrity of the medical images are well preserved.
- Different embedding strategies are designed for different block types, enabling higher payload with an average efficiency of 59%.
- The proposed block classification strategy effectively balances payload and visual quality, achieving an average PSNR of approximately 30 dB.

The remainder of the paper is organized as follows. Section 2 reviews preliminaries. Section 3 presents the proposed method. Section 4 details the experimental results and performance evaluation. Section 5 concludes the paper.

## 2. Preliminaries

This section primarily introduces the two fundamental prerequisite technologies upon which the proposed algorithm relies: the AMBTC compression algorithm as the carrier and the puzzle matrix as the reference matrix.

### 2.1. Absolute Moment Block Truncation Coding (AMBTC)

AMBTC is a classical lossy image compression technique proposed by Lema and Mitchell [6]. The core compression procedure of AMBTC begins by dividing the original image into non-overlapping blocks of size k×k. For each block, the mean pixel value μ is calculated as shown in Equation (1), where $p_{i}$ denotes the grayscale value of the i-th pixel in the current block. After obtaining the mean, each pixel value $p_{i}$ in the block is compared with μ to construct a bitmap BM. If $p_{i}\geq \mu$, the corresponding BM element is assigned 1; otherwise, it is assigned 0. The BM construction rule is defined in Equation (2). Based on the BM, the pixels are classified into a high-value group (marked as 1) and a low-value group (marked as 0). The average grayscale values of these two groups are then computed and denoted as the high quantization value H and the low quantization value L, respectively, as defined in Equations (3) and (4), where q represents the number of pixels with BM value 1 in the block. After these steps, the original k×k pixel block is compressed into a triplet (L,H,BM), which forms the compression representation. During decompression, pixel values are restored according to the BM. Positions marked as 1 are assigned the H, while positions marked as 0 are assigned the L. The reconstructed pixel value ${p^{\prime }}_{i}$ is defined in Equation (5).

(1) $$\mu =\frac{\sum_{i=1}^{k\times k}p_{i}}{k\times k}.$$

(2) $$BM_{i}=\left\{\begin{array}{c} 1,\;p_{i}\geq \mu \\ 0,\;p_{i}<\mu \end{array}\right..$$

(3) $$H=\frac{\sum_{p_{i}\geq \mu }p_{i}}{q}.$$

(4) $$L=\frac{\sum_{p_{i}<\mu }p_{i}}{k\times k-q}.$$

(5) $${p^{\prime }}_{i}=\left\{\begin{array}{c} H,\;BM_{i}=1\\ L,\;BM_{i}=0 \end{array}\right..$$

**Figure 2:**
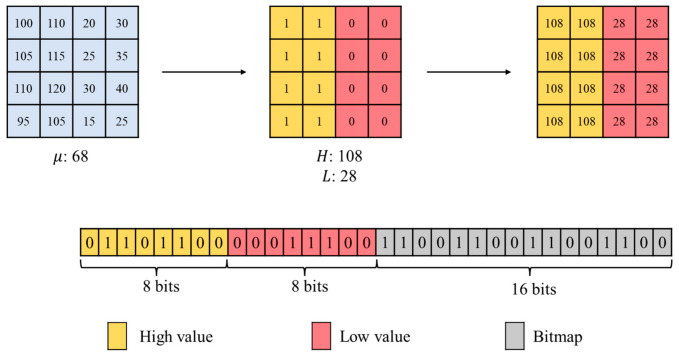
Example of the AMBTC process.

As illustrated in Figure 2, the process of AMBTC is demonstrated using an 4×4 image block. The left side shows the original block with a mean μ=68. The center illustrates the BM obtained by comparing each pixel with the mean. Based on the partition, the high and low quantization values are computed as H=108 and L=28, respectively. The compressed representation consists of H, L, and the corresponding BM. The right side shows the reconstruction stage, where pixel values are replaced with H or L according to the BM, producing an approximated image block.

### 2.2. Puzzle Matrix

In 2025, Lin et al. [29] proposed a dual-image reversible data hiding method using a reference matrix called the puzzle matrix. The matrix is 256×256, corresponding to the pixel value range 0 to 255 of an 8-bit grayscale image. The row index x and column index y represent the grayscale values of two pixels (x,y).

**Figure 3:**
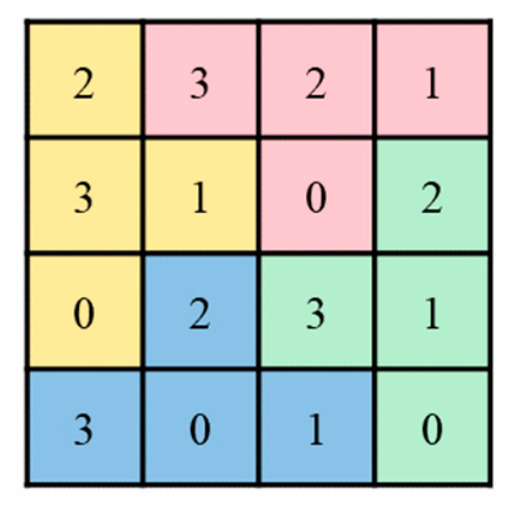
Diagram of the puzzle block.

**Figure 4:**
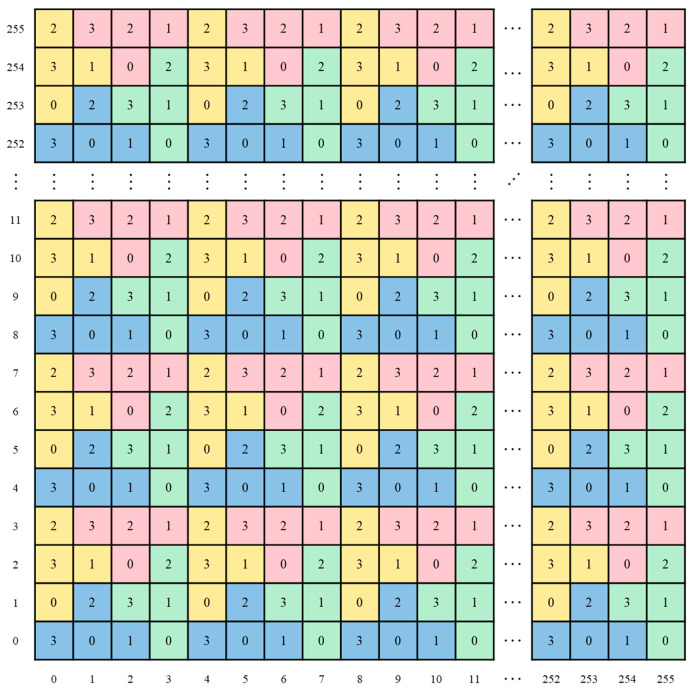
Diagram of the puzzle matrix.

A 4×4 puzzle block is illustrated in Figure 3. This block is partitioned into four puzzle pieces (red, yellow, blue, and green), which are assigned the values 0, 1, 2, and 3 in a counterclockwise order. As shown in Figure 4, the designed 4×4 puzzle block is then replicated and tiled horizontally and vertically until a complete 256×256 matrix is formed.

In this paper, the puzzle matrix is used solely as a reference matrix, and the embedding strategy is independently designed. To preserve visual quality, the method employs a closest-distance principle, limiting pixel modifications to the minimum Euclidean distance.

**Figure 5:**
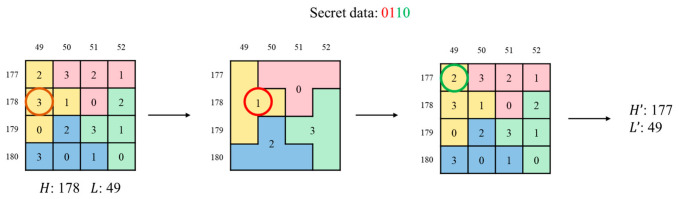
An example of data embedding using the puzzle matrix.

During embedding, H and L are used as the vertical and horizontal coordinates in the matrix. The 4-bit secret data is divided into two 2-bit groups. The first group determines the puzzle piece, and the second specifies the value within it. The target element is then located, and the original coordinate pair is updated to $\left(H^{\prime },L^{\prime }\right)$. As illustrated in Figure 5, assume the AMBTC-quantized levels of an input block are H=178 and L=49, and the secret data is S=0110. The 4-bit secret data S is divided into two 2-bit groups, which yield decimal values 1 and 2. The algorithm identifies the puzzle piece labeled 1 (yellow) and locates the element with value 2 within it. The resulting coordinates (177,49) are assigned to $\left(H^{\prime },L^{\prime }\right)$, successfully embedding the 4-bit secret data.

## 3. Proposed Method

**Figure 6:**
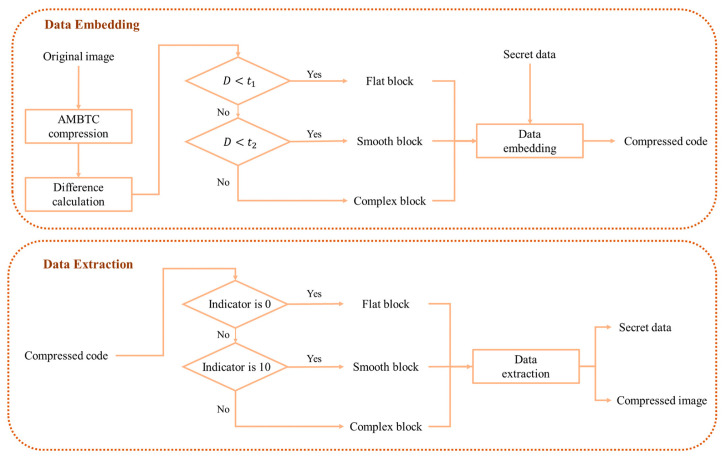
Flowchart of the proposed method.

In this section, we propose an adaptive high-payload data hiding method in AMBTC-compressed medical images. The overall process is illustrated in Figure 6, detailing the embedding and extraction of secret data. Based on the difference D between H and L within a block, image blocks are classified into three categories: flat, smooth, and complex. Customized embedding strategies are applied to each category to maximize space and maintain image quality. Flat and smooth blocks use the mean replacement and dimensionality reduction with a voting principle, respectively, to eliminate BM redundancy, while complex blocks employ a weighted puzzle matrix mechanism to minimize visual distortion. During extraction, identifying each block’s type and applying the corresponding reverse operations enables recovery of both the secret data and the compressed image. Section 3.1, Section 3.2 and Section 3.3 provide detailed descriptions of block classification, data embedding, and data extraction.

### 3.1. Block Classification

This paper uses the AMBTC algorithm for image block classification. Based on the difference D between H and L, image blocks are divided into three categories: flat, smooth, and complex blocks. To properly classify image blocks, we define thresholds $t_{1}=16$ and $t_{2}=32$. The classification can be formally expressed as Equation (6).

(6) $$Block\;type=\left\{\begin{array}{c} \mathrm{flat},\;D\in [0,t_{1})\\ \mathrm{smooth},\;D\in [t_{1},t_{2})\\ \mathrm{complex},\;D\in [t_{2},255] \end{array}\right..$$

### 3.2. Data Embedding

In the data embedding stage, flat blocks store only the block mean, smooth blocks compress the BM using a voting principle, and complex blocks preserve the full BM while applying a puzzle matrix for coordinate mapping and sequence adjustments. Each strategy is designed to balance data payload with visual quality.

**Figure 7:**
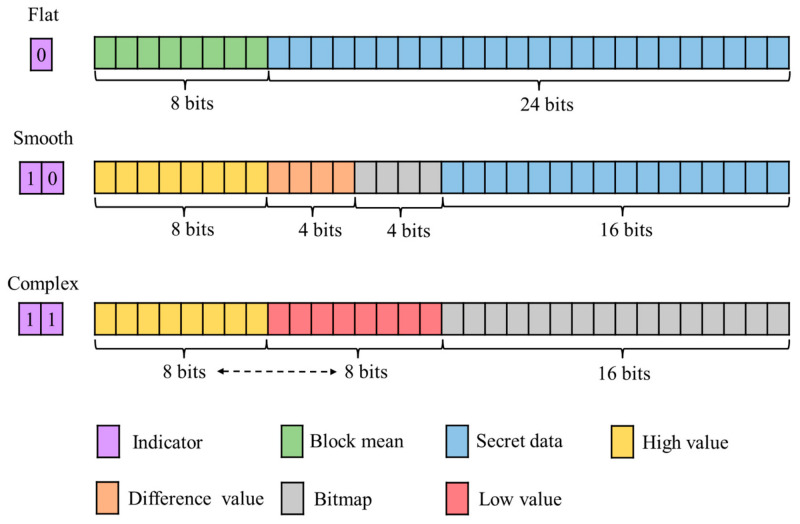
Storage structure for each block type.

The storage structure for each block type is shown in Figure 7. Each type is guided by an indicator, which is used to correctly parse the subsequent data segments. The flat block corresponds to the first row, with an indicator 0. This is followed by an 8-bit block mean μ, and the remaining 24 bits carry secret data. The smooth block corresponds to the second row, with an indicator 10. Its data segments include 8-bit H, $\log_{2}\left(t_{2}-t_{1}\right)$ bits $D^{\prime }$, which under the threshold settings in this example, is equal to $\log_{2}\left(32-16\right)=4$ bits and a 4-bit BM, reserving 16 bits for secret data. The complex block corresponds to the third row, with an indicator 11. Its main body contains 8-bit H, 8-bit L, and the full 16-bit BM. Notably, the dashed double-headed arrow between H and L indicates that their storage order is variable. By swapping H and L, an additional bit of secret data can be embedded. The remaining 4-bit secret data are embedded using the puzzle matrix. Because block types are often spatially continuous, run-length encoding is an effective approach for compressing the indicators, and extended run-length encoding (ERLE) [30] is applied in this paper.

#### 3.2.1. Flat Block Embedding

Blocks classified as flat blocks are assigned an indicator 0. Rather than being embedded within the image, these indicators are additionally stored in the compressed code. Because D is very small, a flat block is represented solely by its block mean, which occupies 8 bits. Therefore, the payload for embedding secret data in a flat block is 32−8=24 bits.

**Figure 8:**
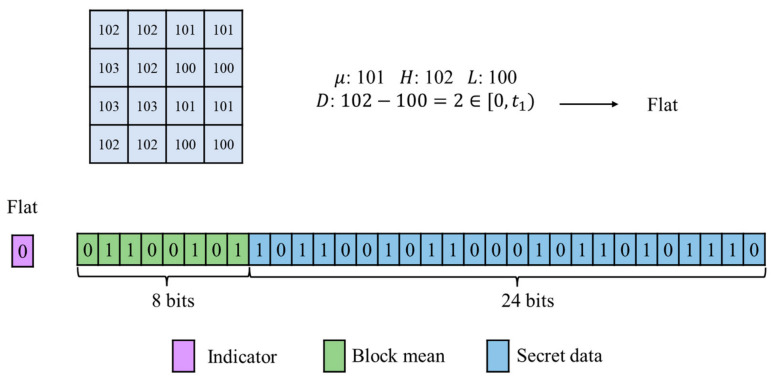
Example of flat block embedding.

Figure 8 illustrates an example of flat block embedding. First, statistical calculations are performed on a 4×4 image block, yielding μ=101, H=102, and L=100. The difference D=H−L=2 is then computed. Since $D\in [0,t_{1})$, the block is classified as flat. In the compression representation, the indicator bit is set to 0, followed by the 8-bit block mean μ. The remaining 24 bits are used to store the secret data.

#### 3.2.2. Smooth Block Embedding

Blocks classified as smooth blocks are assigned an indicator 10. Compared to flat blocks, smooth blocks exhibit more variation and therefore require storing more intrinsic image information. The first component to be preserved is the value of H, which occupies 8 bits. Next, D is stored, requiring $\log_{2}\left(t_{2}-t_{1}\right)$ bits. Under the parameter settings of this paper, this equals $\log_{2}\left(32-16\right)=4$ bits. Given the relationship D=H−L, the value of L can be derived from H and D, so explicitly storing L is unnecessary, saving embedding space. The 4×4 BM is divided into four 2×2 sub-blocks, and a voting principle is applied to retain a single bit per sub-block. If a tie occurs (two 0s and two 1s), it is resolved based on the total number of 0s and 1s in the 4×4 block. If a tie persists, the bit defaults to 1. If the resulting 4 bits are all 1s or all 0s, the block is reclassified as a flat block to further increase payload. Therefore, the payload for embedding secret data in a smooth block is 32−8−4−4=16 bits.

**Figure 9:**
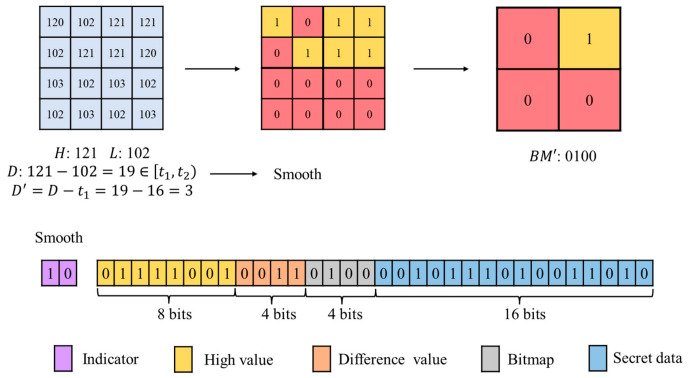
Example of smooth block embedding.

Figure 9 shows a 4×4 smooth block with H=121, L=102, and D=19. Since $D\in [t_{1},t_{2})$, it is classified as smooth. The difference is offset, resulting in $D^{\prime }=D-t_{1}=3$, which is represented as 0011 in 4 bits. The 4×4 BM is divided into four 2×2 blocks, and the voting principle produces a final 2×2 $BM^{\prime }$ of 0100. In the structure, the block type indicator is 10, followed by 8-bit H, 4-bit $D^{\prime }$, and the 4-bit $BM^{\prime }$ from voting. The remaining space is used to embed the secret data.

#### 3.2.3. Complex Block Embedding

Blocks classified as complex blocks are assigned an indicator 11. The intrinsic image information that must be preserved includes the values of H, L, and the BM, which occupy 8 bits, 8 bits, and 16 bits, respectively. H and L are treated as the horizontal and vertical coordinates, respectively, and are mapped onto the puzzle matrix to embed 4-bit secret data. When multiple candidate mappings result in equal modification distances, priority is given to modifying the coordinate with the smaller weight. Specifically, a weighted calculation is performed during mapping to select the position that incurs the minimum overall modification. In the BM, a larger number of 1s indicates a higher weight for H, whereas a larger number of 0s indicates a higher weight for L. In addition, the proposed method embeds one extra bit by swapping the storage positions of $H^{\prime }$ and $L^{\prime }$. In the compressed code, the sequence $\left(H^{\prime },\;L^{\prime },\;BM\right)$ represents 0, whereas $\left(L^{\prime },\;H^{\prime },\;BM\right)$ represents 1. The feasibility of this swapping mechanism is justified as follows. Since the puzzle matrix is composed of periodically tiling 4×4 puzzle blocks containing all 16 possible combinations, a target value matching the secret data can always be found within any 4×4 puzzle block. This means that the maximum adjustment distance of a single-axis coordinate will never exceed |3−0|=3. However, for a block classified as complex, given the parameter setting $t_{2}=32$, the difference D≥32. Therefore, even after the maximum adjustment, the condition $H^{\prime }>L^{\prime }$ always holds. Consequently, the additional bit can be reliably extracted by determining whether the larger value appears first or second in the compressed code.

**Figure 10:**
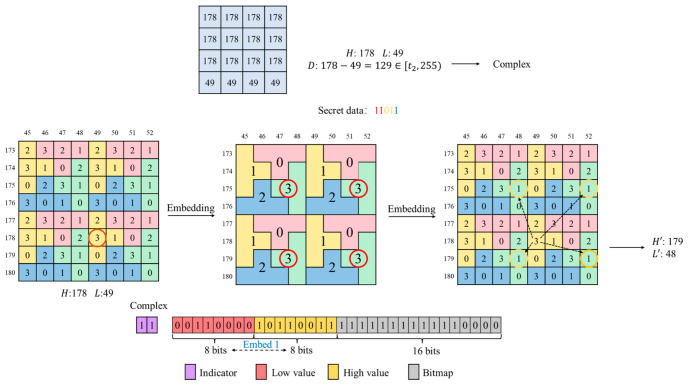
Example of complex block embedding.

As shown in Figure 10, a 4×4 input block yields H=178 and L=49 using the AMBTC method. Since D=129 and $D\in [t_{2},255]$, the block is classified as complex. During embedding, (H,L) is mapped onto the puzzle matrix. The secret data 11 (in red) is converted to 3, locating the corresponding sub-block labeled 3. The secret data 01 (in yellow) is converted to 1, selecting position 1 within that sub-block. Four candidate positions exist; based on the distance calculation to ensure minimal modification, the coordinate (179,48) is selected and denoted as $\left(H^{\prime },L^{\prime }\right)$. The final secret bit 1 (in blue) is embedded by swapping the positions of $H^{\prime }$ and $L^{\prime }$ in the compressed code. Thus, a total of 5-bit secret data is embedded. The final compressed code consists of the indicator 11, followed by the modified 8-bit $L^{\prime }$, 8-bit $H^{\prime }$, and the 16-bit BM.

### 3.3. Data Extraction

This subsection details the process of secret extraction, which essentially reverses the data embedding procedure. The extraction first decompresses the indicators using the ERLE [30] algorithm to identify each block type. Based on the determined type, the corresponding reverse operations are applied to recover the secret data and reconstruct the image blocks.

#### 3.3.1. Flat Block Extraction

During the extraction stage, the indicator of the current image block is first retrieved from the additionally stored information. If the retrieved indicator is 0, the block is identified as a flat block. Since the indicator in the proposed method is not embedded within the image data stream, the receiver directly reads a 32-bit data block sequentially from the compressed stream. According to the AMBTC configuration for flat blocks, the first 8 bits represent the block mean of the intrinsic image information. Consequently, after isolating these initial 8 bits, the remaining 32−8=24 bits constitute the extracted secret data. During image restoration, the receiver directly utilizes the extracted 8-bit block mean to overwrite all pixels within the 4×4 block, thereby completing the reconstruction of this flat block.

**Figure 11:**
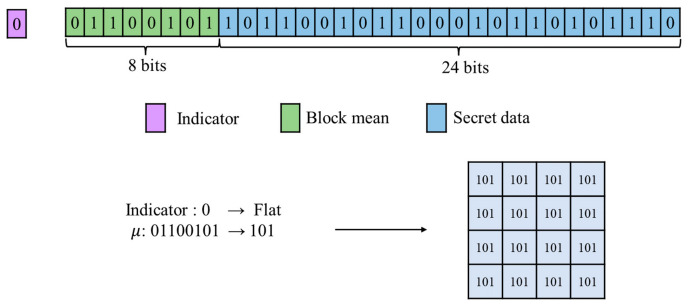
Example of flat block extraction.

As shown in Figure 11, the indicator 0 is first read, confirming that the block is flat. This is followed by 8 bits of block mean, 01100101, which are converted to the decimal value μ=101. Based on the flat block structure, the subsequent 24 bits are directly extracted as embedded secret data. After secret extraction, the recovered mean μ=101 is assigned to all pixels within the 4×4 block, completing reconstruction.

#### 3.3.2. Smooth Block Extraction

If the retrieved indicator is 10, the block is identified as a smooth block. The AMBTC framework is then applied for the extraction process. The first component retrieved is H, occupying 8 bits. Subsequently, $D^{\prime }$ is read. The number of bits required for $D^{\prime }$ is $\log_{2}\left(t_{2}-t_{1}\right)$. Under the parameter settings adopted in this paper, $D^{\prime }$ occupies 4 bits. Given the relationship D=H−L, and the embedding rule $D=D^{\prime }+t_{1}$, the difference D can be recovered. The value of L is then derived using L=H−D. Next, the 4-bit compressed BM is retrieved. During embedding, the 4×4 BM was partitioned into four independent 2×2 sub-blocks and compressed using a voting principle. During extraction, each retrieved bit is replicated four times to reconstruct its corresponding 2×2 sub-block, thereby restoring the complete 4×4 BM. After deducting the intrinsic image information bits, the extracted secret for a smooth block is 32−8−4−4=16 bits. Using the recovered H and L together with the reconstructed 4×4 BM, the image block is restored.

**Figure 12:**
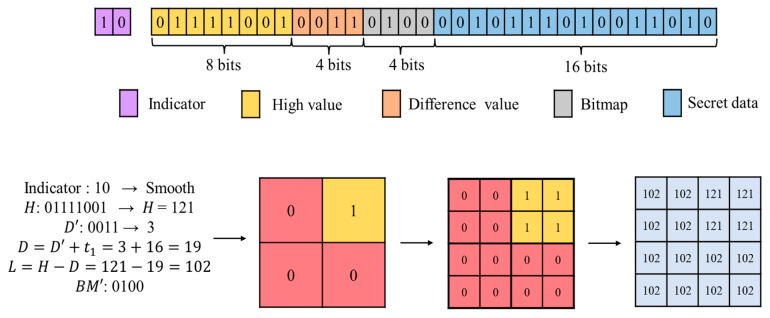
Example of smooth block extraction.

As illustrated in Figure 12, the indicator 10 is identified, and the block is classified as smooth. It then reads 8 bits to obtain H=121. Next, 4-bit $D^{\prime }$ are read, yielding $D^{\prime }=3$. Since $t_{1}=16$ under the adopted parameters, the difference is recovered as $D=D^{\prime }+16=19$. Thus, L=121−19=102. The 4-bit compressed BM is subsequently extracted. The remaining 16 bits in the compressed code are identified as embedded secret data. During reconstruction, the 4-bit BM is expanded into a 4×4 structure. Pixels corresponding to 0 in the BM are assigned L=102, while pixels corresponding to 1 are assigned H=121, completing reconstruction of the 4×4 block.

#### 3.3.3. Complex Block Extraction

If the retrieved indicator is 11, the block is identified as a complex block. During the extraction stage, the first 8 bits, the subsequent 8 bits, and the final 16 bits representing the BM are sequentially read from the compressed stream. The extraction procedure for the information implicitly embedded within H and L is described as follows. Because the block was classified as complex during the embedding stage, the parameter setting $t_{2}=32$ guarantees that the difference satisfies D≥32. Moreover, since the maximum deviation introduced by the puzzle matrix mapping is at most 3, the inequality $H^{\prime }>L^{\prime }$ always holds after modification. Therefore, the relative order of the two retrieved 8-bit values can be reliably used to extract one bit of secret data. Specifically, if the first 8-bit value is greater than the second 8-bit value, the sequence is interpreted as $H^{\prime }$, $L^{\prime }$, followed by the BM, and the extracted secret bit is 0. Conversely, if the first 8-bit value is smaller than the second, the sequence is interpreted as $L^{\prime }$, $H^{\prime }$, followed by the BM, and the extracted secret bit is 1. After restoring the correct coordinate order, $H^{\prime }$ is treated as the horizontal coordinate and $L^{\prime }$ as the vertical coordinate. By mapping the coordinate pair $\left(H^{\prime },L^{\prime }\right)$ onto the same puzzle matrix used during embedding, the corresponding matrix value is obtained, thereby extracting the remaining 4-bit secret data. Using the restored $H^{\prime }$ and $L^{\prime }$ together with the retrieved 16-bit BM, the AMBTC reconstruction procedure is applied to recover the image block. Accordingly, the total amount of secret data extracted from a complex block is 1+4=5 bits.

**Figure 13:**
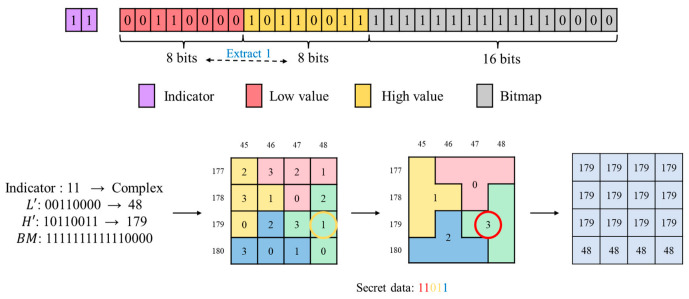
Example of complex block extraction.

As illustrated in Figure 13, the indicator 11 is identified, and the block is classified as complex. It then reads the subsequent two 8-bit values. By determining their order in the compressed code, whether the positions of $H^{\prime }$ and $L^{\prime }$ were swapped is established, allowing one secret bit to be extracted and simultaneously restoring the coordinate pair $\left(H^{\prime },L^{\prime })=(179,48\right)$. Next, mapping this coordinate onto the puzzle matrix locates the corresponding sub-block labeled 3. The decimal value 3 corresponds to 11, yielding 2-bit secret data. Within this sub-block, the cell value at the specified coordinate is 1, which corresponds to 01, yielding an additional 2-bit secret data. At this stage, all 5 bits of secret data have been successfully extracted. The 16-bit BM is subsequently extracted. During reconstruction, pixels corresponding to 0 in the BM are assigned $L^{\prime }=48$, while pixels corresponding to 1 are assigned $H^{\prime }=179$, completing reconstruction of the 4×4 block.

## 4. Experimental Results

### 4.1. Experimental Setup

**Figure 14:**
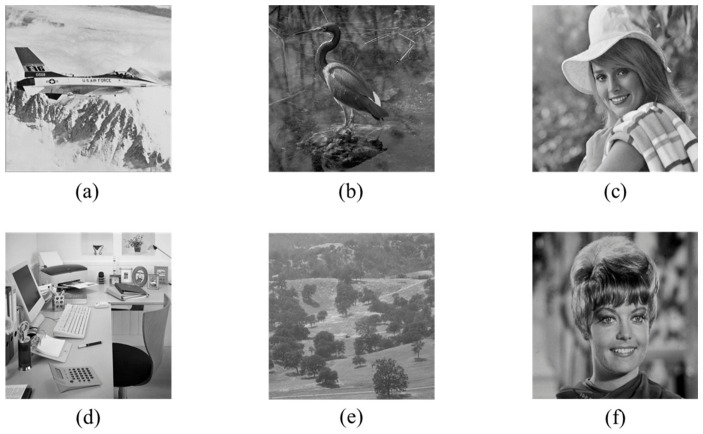
Standard images: (**a**) Airplane, (**b**) Egretta, (**c**) Elaine, (**d**) Office, (**e**) Woodland, (**f**) Zelda.

**Figure 15:**
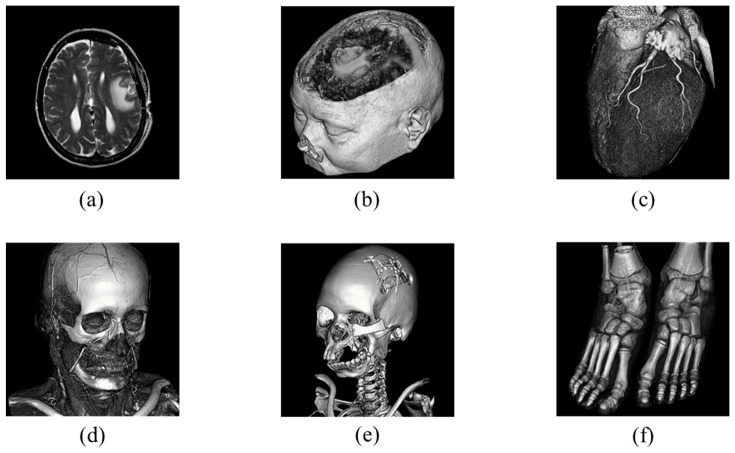
Medical images: (**a**) Brainix, (**b**) Cerebrix, (**c**) Goudurix, (**d**) Manix, (**e**) Phenix, (**f**) Vix.

This subsection presents an experimental evaluation of the proposed method. All experiments were conducted on a Windows 11 laptop (MECHREVO Aurora X (Zhitong Intelligent Technology (Suzhou) Co., Ltd., Suzhou, China)) equipped with an Intel(R) Core(TM) i7-14650HX 2.20 GHz processor and 16 GB of RAM, and the algorithms were implemented in MATLAB R2024b. Six standard grayscale images and six medical grayscale images [31], each of size 512 × 512, were used for testing. The standard images are shown in Figure 14, and the medical images are shown in Figure 15.

The performance of the proposed method is evaluated using peak signal-to-noise ratio (PSNR), structural similarity index (SSIM), payload, and efficiency.

PSNR is commonly used to measure the distortion of the compressed image relative to the original image. A higher PSNR value indicates that the compressed image is closer to the original, indicating better visual quality. The PSNR is defined as shown in Equation (7). The calculation of PSNR depends on the Mean Squared Error (MSE) between two images. The MSE is defined in Equation (8). X(i,j) and $Y\left(i,j\right)$ represent the pixel values at the i row and j column of the two compared images, respectively. The variables m and n denote the height and width of the image. In addition to PSNR, SSIM is adopted to evaluate the structural similarity between two images. SSIM measures the similarity of luminance, contrast, and structural information between images. The SSIM is defined in Equation (9). x and y denote the two image blocks being compared. The variables $\mu_{x}$ and $\mu_{y}$ represent the mean intensities of x and y. The terms ${\sigma_{x}}^{2}$ and ${\sigma_{y}}^{2}$ denote the variances of x and y, while $\sigma_{xy}$ represents the covariance between them. The parameters $C_{1}$ and $C_{2}$ are stabilization constants used to avoid division by zero. The SSIM value ranges from 0 to 1, where a value closer to 1 indicates higher similarity between the two images.

Payload represents the total bits of secret data that can be embedded in the image. Efficiency is defined in Equation (10) as the ratio between the payload and the resulting file size.

(7) $$PSNR=10\times \log_{10}\frac{255^{2}}{MSE}.$$

(8) $$MSE=\frac{1}{m\times n}\sum_{i=1}^{m}\sum_{j=1}^{n}{\left[X\left(i,j\right)-Y(i,j)\right]}^{2}.$$

(9) $$SSIM\left(x,y\right)=\frac{\left(2\mu_{x}\mu_{y}+C_{1}\right)\left(2\sigma_{xy}+C_{2}\right)}{\left({\mu_{x}}^{2}+{\mu_{y}}^{2}+{C_{1}}^{2}\right)\left({\sigma_{x}}^{2}+{\sigma_{y}}^{2}+{C_{2}}^{2}\right)}.$$

(10) $$Efficiency=\frac{Payload}{File\;Size}.$$

### 4.2. Block Classification Analysis

In the proposed method, block classification constitutes the core step that governs the overall payload and visual quality. To intuitively demonstrate the effectiveness of the proposed classification strategy, this subsection conducts an exhaustive statistical and visualization analysis of the block types for the test images.

We tabulated the quantities and corresponding percentages of each block type across the 12 test images under four distinct threshold combinations for comparative testing: (4, 8), (8, 16), (16, 32), and (32, 64). Table 1 illustrates the performance of this classification mechanism under different thresholds for standard images. As $\left(t_{1},t_{2}\right)$ increases, the proportion of flat blocks grows substantially. When the thresholds are relaxed to (32, 64), the proportion of flat blocks in all test images exceeds 85%, with the Woodland and Zelda images reaching 96.50% and 96.77%, respectively. Meanwhile, the proportion of complex blocks remains very low, dropping below 6% for most images. Table 2 further confirms the superior adaptability of this method to medical images. Even at (4, 8), the proportion of flat blocks in half of the medical images exceeds 50%, which is higher than that of standard images. At (32, 64), block classification of medical images reaches an excellent state.

**Figure 16:**
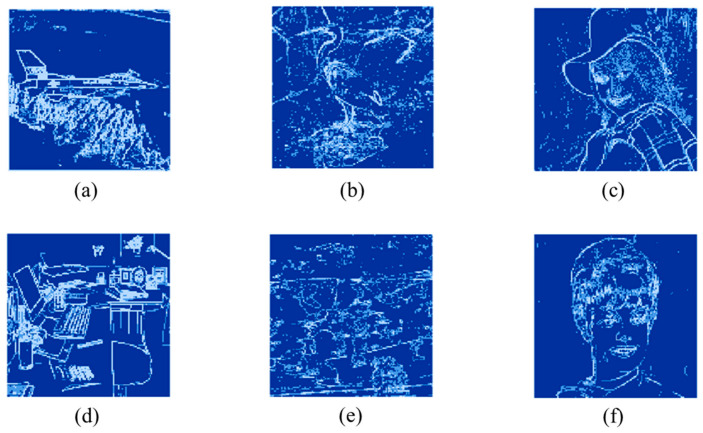
Visualization of block classification for standard images: (**a**) Airplane, (**b**) Egretta, (**c**) Elaine, (**d**) Office, (**e**) Woodland, (**f**) Zelda.

**Figure 17:**
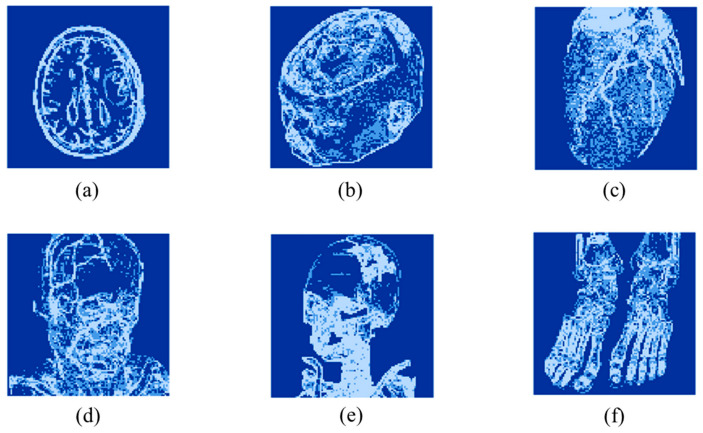
Visualization of block classification for medical images: (**a**) Brainix, (**b**) Cerebrix, (**c**) Goudurix, (**d**) Manix, (**e**) Phenix, (**f**) Vix.

Figure 16 and Figure 17 present the visualization results. Dark blue regions represent blocks classified as flat, blue regions indicate smooth blocks, and light blue regions correspond to complex blocks. Figure 16 illustrates the spatial distribution of block classification for the standard images at a threshold of (16, 32). In Figure 16a Airplane and Figure 16f Zelda, large smooth regions are classified as flat blocks, allowing higher payload. Figure 17 shows the results for a medical image dataset, where images such as Figure 17a Brainix and Figure 17e Phenix contain a high proportion of flat blocks. These visualization results align with Table 2, confirming that the proposed method is well suited for medical images and supports higher payload.

**Figure 18:**
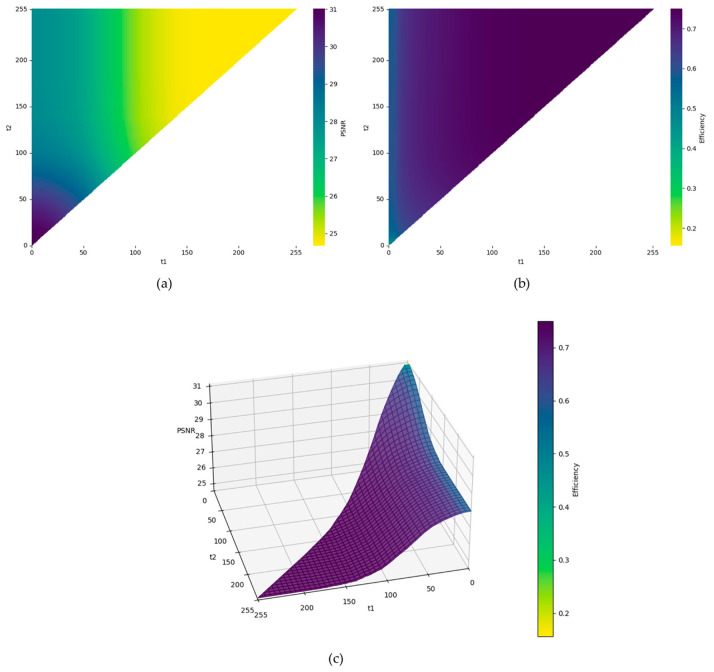
Visualization of the trade-off between image quality and embedding efficiency under different thresholds. (**a**) 2D heatmap of PSNR. (**b**) 2D heatmap of embedding efficiency. (**c**) 3D joint surface plot mapping PSNR to height and efficiency to surface color.

To intuitively analyze the joint impact of the customizable thresholds $t_{1}$ and $t_{2}$, we exhaustively evaluated the PSNR and embedding efficiency across the parameter space. As illustrated in Figure 18, this visualization explicitly demonstrates the inherent trade-off between visual quality and efficiency. Specifically, Figure 18a and Figure 18b present the 2D heatmaps of PSNR and efficiency, respectively, while Figure 18c introduces a 3D surface plot mapping PSNR to height and efficiency to surface color. Regarding the two extreme cases, when the thresholds are set to $\left(t_{1},\;t_{2}\right)=\left(0,\;4\right)$, the system achieves the highest PSNR of 31.01 dB, with a corresponding efficiency of 48.84%. Conversely, setting $\left(t_{1},\;t_{2}\right)=\left(180,\;180\right)$ yields the highest efficiency of 74.99%, but the PSNR significantly degrades to 24.67 dB. Since these two extreme values represent the absolute boundaries of the performance spectrum, relying solely on them is insufficient for evaluating practical application scenarios. Therefore, to provide a smooth observation of the performance transition and facilitate a balanced trade-off, we selected (4, 8), (8, 16), (16, 32), and (32, 64) as the representative threshold pairs for the comparative testing.

### 4.3. Visual and Structural Evaluation

**Figure 19:**
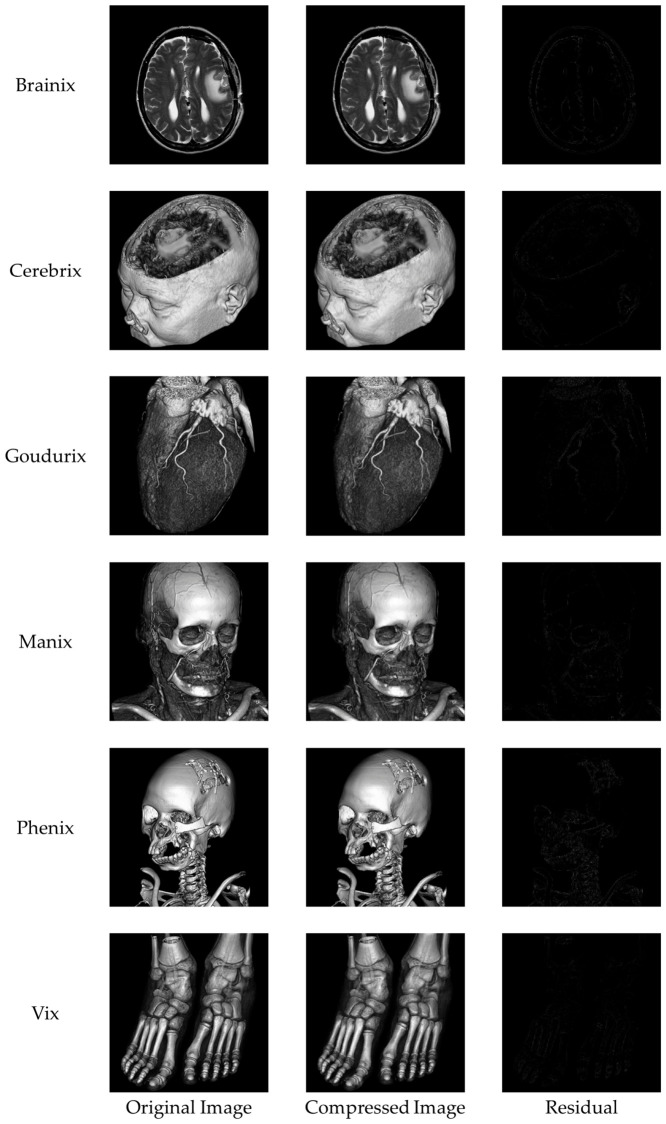
Comparison of the original images, the compressed images, and the residuals.

**Figure 20:**
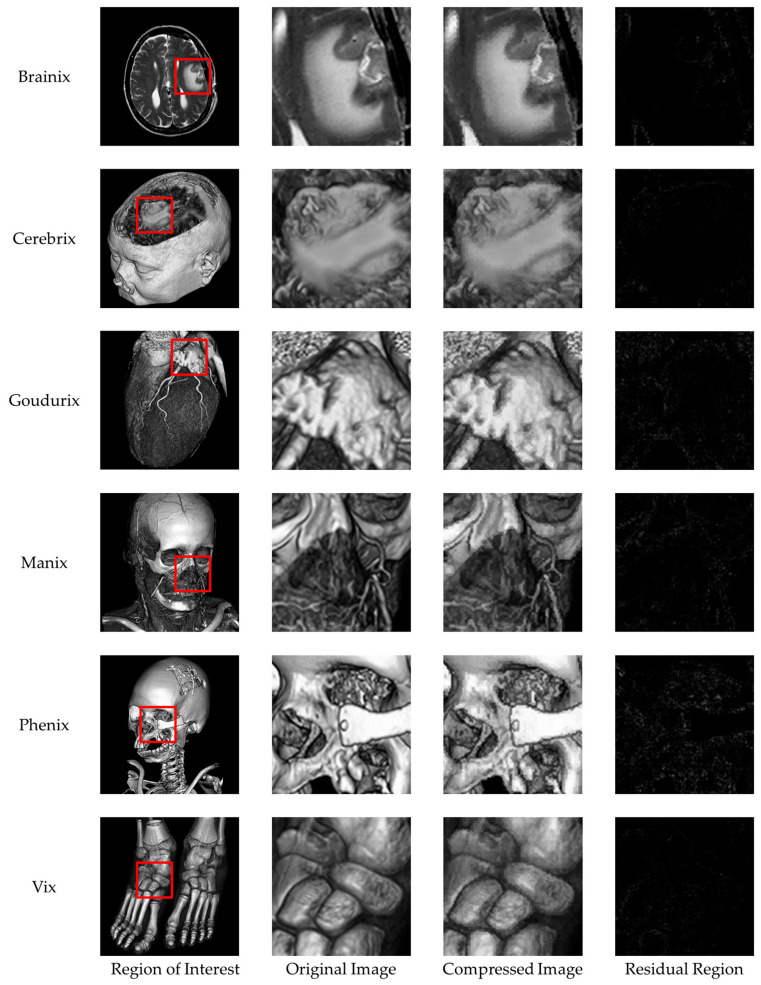
Comparison of ROI in the original images, the compressed images, and the residual regions.

**Figure 21:**
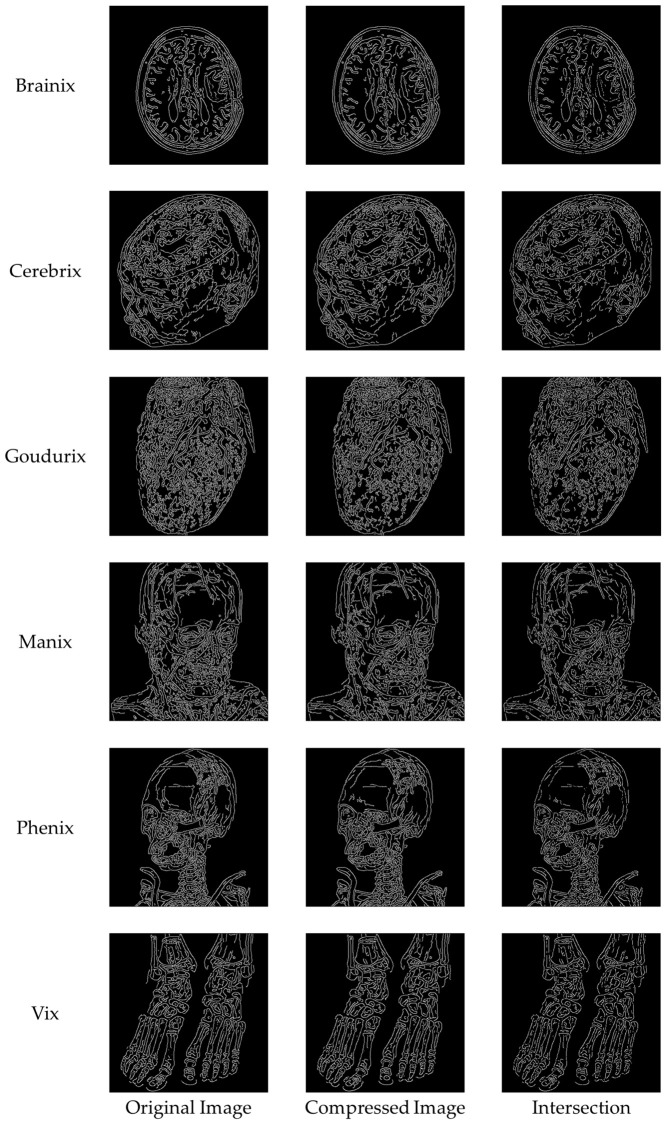
Edge comparison of the original images, the compressed images, and their intersections.

We performed comprehensive visual and structural evaluations to ensure the preservation of critical image features. Figure 19 shows a visual comparison of the original image, the compressed image obtained using the proposed method, and their corresponding residual maps. No significant quality degradation is observed in the compressed image. The residual maps illustrate absolute pixel-level differences and appear almost entirely black across all test images, indicating that the embedding distortion is minimal, uniformly distributed, and imperceptible to the human eye. In medical image analysis, identifying subtle texture variations within the region of interest (ROI) is often essential. Figure 20 shows a magnified view of the ROI marked with a red box. The comparison between the original and reconstructed regions demonstrates that texture, boundaries, and other structural details are well preserved. The corresponding residual maps further confirm that the compression process introduces negligible impact. In addition to pixel-level consistency, maintaining structural and geometric integrity is essential for reliable image quality assessment. Figure 21 evaluates structural preservation using the Canny edge detection method. Edge maps extracted from the original and reconstructed images are compared using intersection maps. The high similarity among these maps indicates strong overlap and continuity of edge structures, suggesting that key boundaries are preserved and the overall morphology remains unchanged.

### 4.4. Security Evaluation

**Figure 22:**
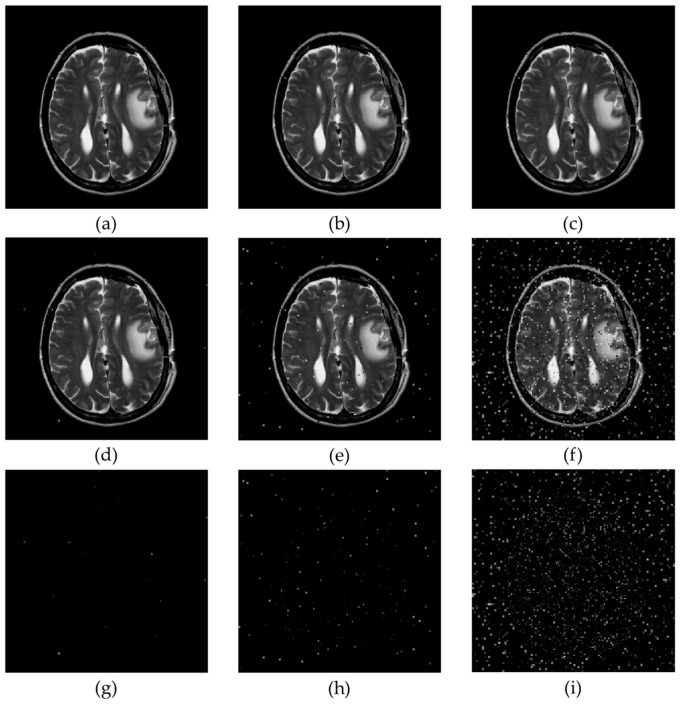
Visual quality comparison of the image Brainix under varying bit-flipping attack intensities. (**a**–**c**) The unattacked images. (**d**–**f**) The attacked images with 100-bit, 1000-bit, and 10,000-bit flips, respectively. (**g**–**i**) The corresponding difference maps between the unattacked and attacked images.

This subsection presents an anti-attack security evaluation. Since the proposed method operates in the compressed domain, the transmitted data consist of compressed codes. We evaluate the robustness of the proposed method against bit-flipping attacks. Specifically, three attack levels are considered, where 100, 1000, and 10,000 bits in the transmitted data are randomly flipped. Figure 22 presents the results for the image Brainix. It can be observed that, because the operations are confined to independent 4 × 4 blocks, corruption of specific compressed codes produces visually localized block-level distortions rather than pixel-level artifacts. Table 3 compares the visual quality and bit error rate (BER) of secret data extraction under different attack conditions. The BER is defined as the ratio of incorrectly extracted secret bits to the total number of embedded secret bits. The results show that when 100 bits are flipped, the average PSNR and SSIM remain high at 39.43 dB and 0.994, respectively. Even when 1000 bits are flipped, the average SSIM remains stable at 0.944. Significant image quality degradation occurs only when 10,000 bits are flipped. From the perspective of secret data extraction, the BER is only 0.03% when 100 bits are flipped, and even when 10,000 bits are flipped, the average BER remains as low as 3.13%. These results demonstrate that the secret data still retain a considerable degree of integrity even under attack.

### 4.5. Performance Evaluation

This subsection further evaluates the performance of the proposed algorithm under different threshold settings. Table 4 and Table 5 present PSNR, SSIM, payload, and efficiency for the standard images and the medical images, respectively. Table 4 shows the results for the standard image set. Efficiency increases as the thresholds $\left(t_{1},\;t_{2}\right)$ are relaxed. At the highest thresholds of (32,64), efficiency exceeds 67% for all images, with Zelda reaching 73.37%. These results indicate that the algorithm effectively utilizes the compressed code to carry secret data. Although higher embedding rates reduce visual quality, the proposed method maintains a PSNR of approximately 30 dB at (32,64). Table 5 presents the performance evaluation results of the algorithm on a medical image dataset. Even at the threshold of (32,64), the SSIM values of medical images are generally higher than those of standard images. For example, the SSIM values of Brainix and Phenix remain 0.909 and 0.892, respectively. Furthermore, Brainix achieves a payload of 222,639 bits with an efficiency of 68.19%.

**Figure 23:**
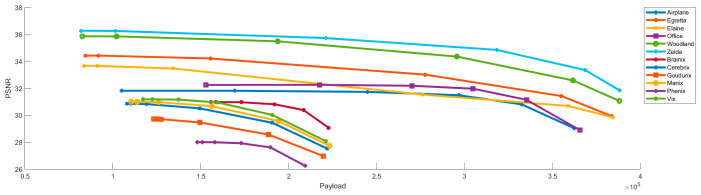
PSNR versus payload under different threshold settings.

**Figure 24:**
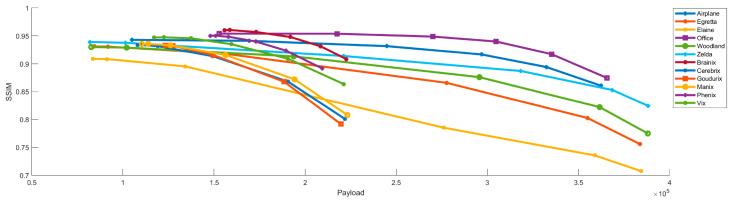
SSIM versus payload under different threshold settings.

The relationships between PSNR, SSIM, and payload under different threshold settings are shown in Figure 23 and Figure 24. Both PSNR and SSIM decrease as payload increases, while most SSIM values remain above 0.9. This behavior reflects the trade-off between visual quality and payload.

To evaluate the performance of the proposed method, comparisons were conducted with several state-of-the-art AMBTC-based data hiding methods [21,22,23,24,25,26,27,28]. All methods were evaluated on the same test images using PSNR, payload, and efficiency. Table 6 presents the comparison results, with the proposed method set at a threshold of (16,32). Methods [21,22,23,24] achieved PSNR values between approximately 26 and 32 dB, with efficiencies not exceeding 43%. Lin et al. [25] focused on high payload and achieved efficiencies above 55%. However, the proposed method outperformed it in PSNR, payload, and efficiency, achieving the highest payload and efficiency across all test images, with an average efficiency of 59%, reaching 63.61% on Brainix. Methods [26,27,28] prioritized visual quality, achieving PSNR around 30 dB and efficiencies above 37%, but with lower payload than the proposed method. Overall, while all methods involve a trade-off between visual quality and payload, the proposed method demonstrates a clear advantage in payload and efficiency while maintaining competitive visual quality.

Table 7 presents a comprehensive comparison with state-of-the-art methods. The components of each method are mainly based on different combinations of block types and embedding strategies. Methods [21,24] use two block types, which provide a simple and clear structure, but their embedding flexibility and payload are limited, representing the main disadvantages of these methods. In contrast, other methods employ three or four block types and can achieve higher payloads by incorporating techniques such as matrix encoding and matrix mapping, which constitute their main advantages in terms of payload. Nevertheless, high-payload methods often require extra file size, which is used to store extra indicators. Method [25] provides two variants: one maintains the original file size without introducing extra overhead, while the other slightly increases the file size to improve payload. Methods [26,27,28] and the proposed method all adopt extra file size to achieve higher payloads. Since AMBTC is a lightweight compression scheme, the computational complexity of all methods is O(n), demonstrating the practicality of AMBTC-based methods in real-world applications. Overall, these methods are designed with different trade-offs among visual quality, payload, and extra file size, and each method exhibits its own advantages depending on specific application scenarios.

## 5. Conclusions

This paper proposes a data hiding method for AMBTC-compressed images in IoMT environments. Image blocks are classified into flat, smooth, and complex categories based on predefined thresholds, and distinct data embedding strategies are applied to each block type. Experimental results demonstrate that the proposed method achieves competitive payload and embedding efficiency compared with state-of-the-art methods, with an average embedding efficiency of 59%, while maintaining PSNR values of approximately 30 dB. Crucially, visual and structural evaluations demonstrate that the proposed method effectively preserves textures and boundaries in medical images. These results confirm the effectiveness and practical applicability of the method for high-payload data hiding in resource-constrained IoMT environments. Future work will investigate adaptive threshold optimization to further enhance data hiding performance in dynamic IoMT scenarios.

## Figures and Tables

**Table 1:** Block classification analysis of standard images.

Thresholds	Block Types	Airplane	Egretta	Elaine	Office	Woodland	Zelda
(4,8)	flat	5953	2046	1210	8560	2029	2428
		(36.33%)	(12.49%)	(7.39%)	(52.25%)	(12.38%)	(14.82%)
	smooth	3827	2681	2259	1959	5632	7165
		(23.36%)	(16.36%)	(13.79%)	(11.96%)	(34.38%)	(43.73%)
	complex	6604	11,657	12,915	5865	8723	6791
		(40.31%)	(71.15%)	(78.83%)	(35.80%)	(53.24%)	(41.45%)
(8,16)	flat	10,019	6451	5538	10,907	8158	9855
		(61.15%)	(39.37%)	(33.80%)	(66.57%)	(49.79%)	(60.15%)
	smooth	2040	6101	7404	1302	4888	4093
		(12.45%)	(37.24%)	(45.19%)	(7.95%)	(29.83%)	(24.98%)
	complex	4325	3832	3442	4175	3338	2436
		(26.40%)	(23.39%)	(21.01%)	(25.48%)	(20.37%)	(14.87%)
(16,32)	flat	12,280	12,941	13,177	12,623	13,272	14,108
		(74.95%)	(78.99%)	(80.43%)	(77.04%)	(81.01%)	(86.11%)
	smooth	1560	2470	2419	1220	2501	1688
		(9.52%)	(15.08%)	(14.76%)	(7.45%)	(15.26%)	(10.30%)
	complex	2544	973	788	2541	611	588
		(15.53%)	(5.94%)	(4.81%)	(15.51%)	(3.73%)	(3.59%)
(32,64)	flat	14,057	15,496	15,615	14,303	15,811	15,854
		(85.80%)	(94.58%)	(95.31%)	(87.30%)	(96.50%)	(96.77%)
	smooth	1328	739	569	1199	569	498
		(8.11%)	(4.51%)	(3.47%)	(7.32%)	(3.47%)	(3.04%)
	complex	999	149	200	882	4	32
		(6.10%)	(0.91%)	(1.22%)	(5.38%)	(0.02%)	(0.20%)

**Table 2:** Block classification analysis of medical images.

Thresholds	Block Types	Brainix	Cerebrix	Goudurix	Manix	Phenix	Vix
(4,8)	flat	5889	3279	3969	3485	5397	3985
		(58.89%)	(32.79%)	(39.69%)	(34.85%)	(53.97%)	(39.85%)
	smooth	859	528	151	763	415	890
		(8.59%)	(5.28%)	(1.51%)	(7.63%)	(4.15%)	(8.90%)
	complex	3252	6193	5880	5752	4188	5125
		(32.52%)	(61.93%)	(58.80%)	(57.52%)	(41.88%)	(51.25%)
(8,16)	flat	6856	4014	4314	4474	5917	4994
		(68.56%)	(40.14%)	(43.14%)	(44.74%)	(59.17%)	(49.94%)
	smooth	969	1925	1452	1775	867	1219
		(9.69%)	(19.25%)	(14.52%)	(17.75%)	(8.67%)	(12.19%)
	complex	2175	4061	4234	3751	3216	3787
		(21.75%)	(40.61%)	(42.34%)	(37.51%)	(32.16%)	(37.87%)
(16,32)	flat	7927	6128	5931	6437	6885	6373
		(79.27%)	(61.28%)	(59.31%)	(64.37%)	(68.85%)	(63.73%)
	smooth	707	2203	2344	1986	795	1778
		(7.07%)	(22.03%)	(23.44%)	(19.86%)	(7.95%)	(17.78%)
	complex	1366	1669	1725	1577	2320	1849
		(13.66%)	(16.69%)	(17.25%)	(15.77%)	(23.20%)	(18.49%)
(32,64)	flat	8721	8403	8358	8535	7833	8225
		(87.21%)	(84.03%)	(83.58%)	(85.35%)	(78.33%)	(82.25%)
	smooth	694	1219	1081	1100	1047	1489
		(6.94%)	(12.19%)	(10.81%)	(11.00%)	(10.47%)	(14.89%)
	complex	585	378	561	365	1120	286
		(5.85%)	(3.78%)	(5.61%)	(3.65%)	(11.20%)	(2.86%)

**Table 3:** Comparison of visual quality and secret data bit error rate under different flip attack intensities.

Images	100 bits			1000 bits			10,000 bits		
	PSNR	SSIM	BER	PSNR	SSIM	BER	PSNR	SSIM	BER
Brainix	38.23	0.993	0.03%	29.52	0.941	0.29%	19.96	0.554	3.14%
Cerebrix	40.51	0.993	0.03%	29.48	0.947	0.30%	19.50	0.576	3.13%
Goudurix	42.70	0.997	0.03%	30.08	0.947	0.33%	19.66	0.576	3.09%
Manix	37.59	0.992	0.03%	29.07	0.938	0.32%	19.64	0.568	3.12%
Phenix	37.98	0.992	0.03%	28.61	0.941	0.31%	19.44	0.568	3.17%
Vix	39.55	0.994	0.03%	29.78	0.950	0.32%	19.66	0.570	3.15%

**Table 4:** Comparison of six standard images at different thresholds.

Images	Airplane	Egretta	Elaine	Office	Woodland	Zelda
Thresholds (4,8)						
PSNR (dB)	31.74	34.22	33.48	32.20	35.49	35.73
SSIM	0.932	0.918	0.896	0.949	0.914	0.914
File Size (bits)	545,553	547,800	541,673	542,082	552,965	554,747
Payload (bits)	244,778	155,647	134,277	270,027	193,687	221,197
Efficiency (%)	44.87	28.41	24.79	49.81	35.03	39.87
Thresholds (8,16)						
PSNR (dB)	31.50	33.02	31.54	31.99	34.36	34.86
SSIM	0.917	0.866	0.786	0.940	0.876	0.887
File Size (bits)	540,741	553,666	555,817	540,554	550,345	546,503
Payload (bits)	296,761	277,701	275,990	304,777	295,578	318,281
Efficiency (%)	54.88	50.16	49.65	56.38	53.71	58.24
Thresholds (16,32)						
PSNR (dB)	30.82	31.44	30.70	31.16	32.60	33.36
SSIM	0.894	0.803	0.736	0.917	0.822	0.853
File Size (bits)	538,371	540,575	540,415	539,076	539,439	535,303
Payload (bits)	332,400	354,969	358,892	335,177	361,599	368,540
Efficiency (%)	61.74	65.67	66.41	62.18	67.03	68.85
Thresholds (32,64)						
PSNR (dB)	29.05	29.95	29.86	28.90	31.07	31.86
SSIM	0.861	0.756	0.708	0.875	0.775	0.825
File Size (bits)	535,146	530,859	531,324	535,483	529,208	529,028
Payload (bits)	362,283	383,734	384,295	365,667	388,019	388,126
Efficiency (%)	67.70	72.29	72.33	68.29	73.32	73.37

**Table 5:** Comparison of six medical images at different thresholds.

Images	Brainix	Cerebrix	Goudurix	Manix	Phenix	Vix
Thresholds (4,8)						
PSNR (dB)	30.98	30.84	29.72	30.99	28.01	31.17
SSIM	0.957	0.932	0.933	0.933	0.949	0.946
File Size (bits)	328,149	326,904	323,973	327,489	325,706	328,574
Payload (bits)	173,058	119,165	127,374	126,134	157,938	137,285
Efficiency (%)	52.74	36.45	39.32	38.52	48.49	41.78
Thresholds (8,16)						
PSNR (dB)	30.85	30.52	29.48	30.68	27.94	30.96
SSIM	0.948	0.914	0.916	0.916	0.940	0.935
File Size (bits)	328,482	332,749	330,824	332,157	327,357	331,224
Payload (bits)	191,892	149,366	149,390	156,306	172,827	159,514
Efficiency (%)	58.42	44.89	45.16	47.06	52.79	48.16
Thresholds (16,32)						
PSNR (dB)	30.38	29.45	28.58	29.58	27.63	30.04
SSIM	0.932	0.868	0.868	0.872	0.924	0.909
File Size (bits)	327,615	332,668	332,120	331,942	327,870	331,838
Payload (bits)	208,390	190,665	188,473	194,149	189,560	190,645
Efficiency (%)	63.61	57.31	56.75	58.49	57.82	57.45
Thresholds (32,64)						
PSNR (dB)	29.09	27.54	26.99	27.74	26.26	28.09
SSIM	0.909	0.801	0.792	0.808	0.892	0.864
File Size (bits)	326,520	327,827	327,559	327,909	327,707	328,614
Payload (bits)	222,639	221,847	219,612	223,165	209,297	221,165
Efficiency (%)	68.19	67.67	67.05	68.06	63.87	67.30

**Table 6:** Performance comparison with state-of-the-art methods.

Methods	Images	Airplane	Brainix	Cerebrix	Goudurix	Manix	Phenix	Vix
[21]	PSNR (dB)	31.71	30.95	30.83	29.75	30.97	28.01	31.17
	Payload (bits)	174,754	117,580	74,950	75,400	82,000	101,635	88,390
	Efficiency (%)	33.33	36.74	23.42	23.56	25.63	31.76	27.62
[22]	PSNR (dB)	31.33	30.68	29.98	29.02	30.19	27.82	30.48
	Payload (bits)	200,912	130,250	101,608	100,808	106,328	117,118	109,234
	Efficiency (%)	38.32	40.7	31.75	31.5	33.23	36.6	34.14
[23]	PSNR (dB)	29.94	29.59	28.73	27.84	29.02	26.92	29.4
	Payload (bits)	210,142	134,698	110,668	110,122	114,513	123,444	116,316
	Efficiency (%)	40.08	42.09	34.58	34.41	35.79	38.58	36.35
[24]	PSNR (dB)	31.85	31.05	30.96	29.73	31.06	28.04	31.42
	Payload (bits)	209,086	136,810	107,323	99,463	109,795	118,348	115,369
	Efficiency (%)	38.67	41.46	32.52	30.14	33.27	35.86	34.96
[25]	PSNR (dB)	29.13	30.05	28.63	27.7	28.75	27.45	29.35
	Payload (bits)	330,200	205,399	185,092	183,640	189,136	185,581	185,806
	Efficiency (%)	61.42	62.67	55.59	55.26	56.95	56.58	55.96
[26]	PSNR (dB)	32.78	32.06	31.52	30.66	31.68	29.27	32.14
	Payload (bits)	251,041	266,441	210,627	204,022	218,061	235,234	221,083
	Efficiency (%)	46.43	49.28	38.96	37.73	40.33	43.51	40.89
[27]	PSNR (dB)	32.20	33.69	34.95	37.29	37.97	N/A	N/A
	Payload (bits)	242,725	267,687	282,648	291,183	296,591	N/A	N/A
	Efficiency (%)	44.56	49.23	52.03	53.71	54.7	N/A	N/A
[28]	PSNR (dB)	32.20	32.50	31.91	31.17	32.16	30.01	32.44
	Payload (bits)	242,725	264,921	222,450	222,655	227,308	240,457	229,475
	Efficiency (%)	44.56	48.72	40.76	40.77	41.66	44.09	42.03
Proposed	PSNR (dB)	30.82	30.38	29.45	28.58	29.58	27.63	30.04
	Payload (bits)	332,400	208,390	190,665	188,473	194,149	189,560	190,645
	Efficiency (%)	61.74	63.61	57.31	56.75	58.49	57.82	57.45

**Table 7:** Comprehensive comparison with state-of-the-art methods.

Methods	Block Types	Key Components	Extra File Size	Computational Complexity
[21]	2	bitmap replacement quantization level exchange	No	O(n)
[22]	3	bitmap replacement Hamming distance calculation pixel value differencing	No	O(n)
[23]	3	bitmap replacement matrix encoding symmetric quantization value embedding	No	O(n)
[24]	2	gradient-based compression bitmap replacement quantization level exchange	No	O(n)
[25]	3	bitmap replacement side match prediction turtle shell matrix mapping quantization level exchange	Optional	O(n)
[26]	4	bitmap replacement matrix encoding turtle shell matrix mapping least significant bit substitution quantization level exchange	Yes	O(n)
[27]	4	bitmap replacement matrix encoding turtle shell matrix mapping least significant bit substitution	Yes	O(n)
[28]	4	bitmap replacement matrix encoding turtle shell matrix mapping least significant bit substitution	Yes	O(n)
Proposed	3	bitmap replacement voting principle puzzle matrix mapping quantization level exchange	Yes	O(n)

## Data Availability

The original contributions presented in this study are included in the article. Further inquiries can be directed to the corresponding authors.
